# Free sugar intake is associated with reduced proportion of circulating invariant natural killer T cells among women experiencing overweight and obesity

**DOI:** 10.3389/fimmu.2024.1358341

**Published:** 2024-05-13

**Authors:** Renad M. Alhamawi, Yahya A. Almutawif, Bushra H. Aloufi, Jory F. Alotaibi, Manar F. Alharbi, Nura M. Alsrani, Razan M. Alinizy, Waad S. Almutairi, Wed A. Alaswad, Hamza M. A. Eid, Walaa A. Mumena

**Affiliations:** ^1^ Department of Clinical Laboratory Sciences, College of Applied Medical Sciences, Taibah University, Madinah, Saudi Arabia; ^2^ Clinical Nutrition Department, College of Applied Medical Sciences, Taibah University, Madinah, Saudi Arabia

**Keywords:** free sugar intake, smoking, physical activity, weight status, central obesity, iNKT cells

## Abstract

**Background:**

Higher prevalence of obesity has been observed among women compared to men, which can be explained partly by the higher consumption of sweets and physical inactivity. Obesity can alter immune cell infiltration, and therefore increase the susceptibility to develop chronic inflammation and metabolic disorders. In this study, we aimed to explore the association between free sugar intake and other unhealthy lifestyle habits in relation to the proportion of circulating iNKT cells among women with healthy weight and women experiencing overweight and obesity.

**Methods:**

A cross-sectional study was conducted on 51 Saudi women > 18 years, wherein their daily free sugar intake was assessed using the validated Food Frequency Questionnaire. Data on smoking status, physical activity, and supplement use were also collected. Anthropometric data including height, weight, waist circumference were objectively measured from each participants. The proportion of circulating iNKT cells was determined using flow cytometry.

**Results:**

Smoking, physical activity, supplement use, and weight status were not associated with proportion of circulating iNKT cells. Significant association was found between proportion of circulating iNKT cells and total free sugar intake and free sugar intake coming from solid food sources only among women experiencing overweight and obesity (Beta: -0.10: Standard Error: 0.04 [95% Confidence Interval: -0.18 to -0.01], *p*= 0.034) and (Beta: -0.15: Standard Error: 0.05 [95% Confidence Interval: -0.25 to -0.05], *p*= 0.005), respectively.

**Conclusion:**

Excessive free sugar consumption may alter iNKT cells and consequently increase the risk for chronic inflammation and metabolic disorders.

## 1 Introduction

The rate of overweight and obesity have increased dramatically since 1990 (1). In 2022, the World Health Organization (WHO) reported a prevalence of overweight and obesity in adults of 43% and 16%, respectively (1). Higher prevalence of obesity has been reported among women compared to men in several settings, including Saudi Arabia (2, 3). Obesity is defined as “the status of accumulation of adipose tissues”, which can affect the function of distinct body systems, including the immune system (4, 5). The enlargement of adipocytes can disrupt immune system homeostasis, increasing the risk for chronic inflammation and several types of cancer (6, 7).

Several factors such as unhealthy lifestyle habits, have been found to be associated with increased risk of obesity and consequently, inflammatory and autoimmune diseases (8, 9). The typical Western diet primarily comprises of processed, packaged and fast foods that contain large amounts of free/added sugar, salt, fat, and limited amounts of healthy food options such as vegetables and fruits (10, 11). This diet has been adopted in many regions in the Middle East along with physical inactivity contributing to the increased incidence of obesity (12–14). Data suggest higher prevalence of physical inactivity and sugar intake among women which can be explained by the hormonal changes experienced monthly by women in reproductive age (14, 15). Additionally, younger women found to be more susceptible to inflammation and infection (16, 17). Accumulating evidence shows that excessive free/added sugar intake directly affects both innate and adaptive immunity (18–21). For example, diet containing high amount of fructose can induce proinflammatory status in mice by increasing neutrophils recruitment and cytokines production (22). T lymphocytes also play a crucial role by exacerbating autoimmunity in animal treating with high level of glucose (23). However, there is a lack of research investigating the link between free sugar consumption and invariant natural killer T (iNKT) cells, a unique population of immune cells, among women.

iNKT cells are an immune subset of immune cells linking both innate and adaptive immunity. The hallmark feature of iNKT cells is the expression of a semi-invariant αβ T cell receptor (TCR) that recognizes glycolipids presented by CD1d molecules (24–26). Although iNKT cells are distinct lineage from conventional αβ T cells, they develop in the thymus and have different subsets (25). Similar to conventional CD4^+^ T cells, iNKT cells can be further divided into different subsets based on their transcription factor expression and pattern of cytokine production pattern: iNKT1, iNKT2, iNKT17 and iNKT10 (27). Accordingly, iNKT cells play a crucial role in both pro-inflammatory and anti-inflammatory responses (28–30). For instance, iNKT1 cells producing IFN-γ is a well-known subset that have a crucial role in anti-tumor and anti-viral immunity while iNKT2 and iNKT17 shown to have immunoregulatory properties (27, 31, 32). iNKT10 cells producing IL-10, on the other hand, are found to be abundant in adipose tissues and associated with immune homeostasis (33). Collectively, these previously published data emphasized the importance iNKT cell subsets in immune responses and homeostasis.

There are limited data exploring the link between free sugar intake and other unhealthy lifestyle habits in relation to proportion of circulating iNKT cells. Thus, we aimed in this study to explore the association between free sugar intake, smoking, physical activity, and supplement use in relation to the proportion of circulating iNKT cells among women with healthy weight and women experiencing overweight and obesity.

## 2 Materials and methods

### 2.1 Study design and population

A cross-sectional study was conducted among adult women recruited from January to March 2023 at Taibah University, Madinah, Saudi Arabia. Women who were < 19 years old, were pregnant, had a medical history of chronic or autoimmune diseases, or had previously received weight management interventions were excluded. The minimum number of participants needed in this study was 38 women based on expected correlation between body mass index (BMI) and proportion of circulating iNKT cells of 0.50, 90% power, and significant level of 95% (34).

Ethical certificate to conduct this study was obtained from the Scientific Research and Ethics Committee of the College of Applied Medical Sciences, Taibah University, Madinah (project number 2023/154/105 MLT). Signed consent form was obtained from all participants included in this study before data collection.

### 2.2 Data collection

Data were collected through face-to-face interviews with the participants using a designed two-part questionnaire to ensure high accuracy of information. The first part of the questionnaire collected sociodemographic data (i.e., age, marital status, employment status, and nationality). Additionally, questions concerning family history of obesity, smoking status, allergies, exercise and other physical activities, dietary plans, weight management interventions, and medical or over-the-counter drugs taken were questioned. Also, participants were asked about supplement use, such as multivitamins, iron, vitamin D, and calcium supplements. Participants were asked to provide data on usual intake of free sugar and then anthropometric measurements and blood sample were collected from each participant.

#### 2.2.1 Assessment of free sugar intake

Food Frequency Questionnaire (FFQ), semi-quantitative, that was designed to assess the intake of free sugar over the past month (35). The WHO defines free sugars as “monosaccharides and disaccharides added to foods and drinks by the manufacturer, cook or consumer, and sugars naturally present in honey, syrups, fruit juices and fruit juice concentrates.” (36). In short, the FFQ included 12 food groups: “Sweetened beverages”; “Ready to eat cereals”; “Bread and rolls”; “Sweet bakery products”; “Quick breads and bread products”; “Candy”; “Other desserts”; “Sugars”; “Yogurt”; “`Mixed dishes”; “Condiments and sauces”; “Fruits” and 41 food items. Frequency options used were: “per month (< once or 1–3 times)”, “per week (once, 2–4 times, or 5–6 times)”, “per day (once, 2–3 times, 4–5 times, or 6 times or more)”. Frequency options were shared with participants to increase the accuracy of data collection. Responses to FFQ for each participant were recorded on a hard copy then entered into an Excel sheet that calculates the content of total free sugar consumed per day in grams. This tool allows for the calculation of free sugar intake coming from solid food sources and free sugar intake coming from liquid food sources. Groups based on the WHO recommendation concerning free sugar intake were later created (free sugar intake within the WHO recommendation [< 25 g/day] vs. free sugar intake exceeded the WHO recommendation [≥ 25 g/day]) (36).

#### 2.2.2 Assessment of anthropometric

The weight (in kg) and height (in cm) of all participants were objectively measured following a standardized procedure using digital scale (Beurer, UK) and stadiometer (Seca, Germany), respectively. Weight and height were used to calculate the BMI for each participant. The WHO cut-offs were used to assess the weight status as follows: “underweight” BMI < 18.5 kg/m^2^; “healthy weight” 18.5 to 24.9 kg/m^2^; “overweight” 25.0 to 29.9 kg/m^2^; “obesity” ≥ 30.0 kg/m^2^ (37, 38). Additionally, the waist circumference (WC) was measured in centimeters where the tape measure placed around the middle at a point halfway between the ribs bottom and hips top just above the belly button. Cutoff used for WC for women is > 88 cm to indicate abdominal obesity (39).

#### 2.2.3 Flow cytometry

Peripheral blood mononuclear cells (PBMCs) and polymorphonuclear cells were isolated from whole-blood samples of the participants collected in EDTA tubes by adding 1 mL of RBC lysing buffer (Thermo Fisher, Massachusetts, USA) for 10 min. Thereafter, the cell suspension was centrifuged at 1500 rpm for 5 min. The cell pellets were initially stained with Live/Dead stain (Thermo Fisher) to exclude all dead cells. The following antibodies from Thermo Fisher were then used to stain the cell pellets to identify the iNKT cells: anti-CD3 (PerCP-CY5.5), anti-CD4 (FITC), and anti-Vα24Jα18 (PE). The stained cell suspension was evaluated using the Attune Flow Cytometer (Thermo Fisher), and the flow cytometry data were analyzed using FlowJo version 10 (LLC, USA).

### 2.3 Statistical analysis

All tests and graphical representations included in this study were conducted using GraphPad Prism version 10 (San Diego, USA) and SPSS version 20 (SPSS, Inc., Chicago, IL, USA). The normality of the distribution of all continuous variables was assessed using the Shapiro–Wilk test. Spearman correlation analysis was performed to assess the strength of association between two continuous variables, whereas the Mann–Whitney test was conducted to compare median values between two different groups. Fisher’s exact test was used to explore the association between two categorical variables. Further, simple linear regression analysis was conducted to investigate the association between total free sugar intake and sugar intake from solid and liquid food sources (independent variable) and the proportion of circulating iNKT cells (dependent variable) adjusting for weight status using stratified analysis. A confidence level of 95% was used to assess significance of the test results.

## 3 Results

### 3.1 Characteristics of the participants

A total of 51 women were included in this study. Over half of them were between 21 to 30 years old (51%, n = 26), and 96.1% (n = 49) were Saudis. The majority of the participants were singles (92.1%, n = 47), and 88.2% (n = 45) were students. About 24% of participants reported a family history of obesity; however, 66.7% (n = 34) of the participants were experiencing overweight and obesity. Further, 41.2% of participants (n = 21) had central obesity, as indicated by a WC of >88 cm. Most women reported consumption of free sugar ≥ 25g/d (92.2%, n= 47). Approximately 12% (n = 6) of participants were smokers, and 19.6% (n = 10) reported using supplements. Twenty-nine percent of the sample reported not to perform any physical activity (n = 15). The characteristics of the participants are presented in details in **Table 1**.

**Table 1:** Sample characteristics (n= 51).

	n	%
Age		
≤ 20 years	22	43.1
21-30 years	26	51.0
> 31 years	3	5.90
Nationality		
Saudi	49	96.1
Non-Saudi	2	3.90
Marital status		
Single	47	92.1
Married	3	5.90
Divorced	1	2.00
Employment status		
Student	45	88.2
Employed	2	3.90
Unemployed	4	7.90
Family history of obesity		
Yes	12	23.5
No	39	76.5
Weight status		
Healthy weight (BMI: 18.5 – 24.9 kg/m^2^)	17	33.3
Overweight (BMI: 25.0 – 29.9 kg/m^2^)	15	29.4
Obese (BMI: ≥ 30.0 kg/m^2^)	19	37.3
Waist circumference		
≤ 88 cm	30	58.8
> 88 cm	21	41.2
Free sugar intake		
< 25 g/d	4	7.80
≥ 25 g/d	47	92.2
Smoking status		
Yes	6	11.8
No	45	88.2
Supplement use		
Yes	10	19.6
No	41	80.4
Physical activity		
None	15	29.4
Once per week	9	17.6
2-3 times per week	18	35.3
4-5 times per week	1	1.96
Daily	8	15.7

### 3.2 Association between characteristics of the study sample and weight status

**Table 2** illustrates the characteristics of the study sample stratified by weight status. Significantly higher proportion of women experiencing overweight and obesity have WC > 88 cm compared to women with healthy weight (100% vs. 0.00%, *p* < 0.001, respectively). All other characteristics of the sample were similar across the different weight status groups.

**Table 2:** Characteristics of the study sample stratified by weight status (n= 51).

	Healthy weight (n= 17)	Overweight and obese (n= 34)	*p-value*
Age			
≤ 20 years	9 (40.9)	13 (59.1)	0.642
21-30 years	7 (26.9)	19 (73.1)	
> 31 years	1 (33.3)	2 (66.7)	
Nationality			
Saudi	16 (32.7)	33 (67.3)	1.00
Non-Saudi	1 (50.0)	1 (50.0)	
Marital status			
Single	17 (36.2)	30 (63.8)	0.695
Married	0 (0.00)	3 (100)	
Divorced	0 (0.00)	1 (100)	
Employment status			
Student	16 (35.6)	29 (64.4)	0.813
Employed	0 (0.00)	2 (100)	
Unemployed	1 (25.0)	3 (75.0)	
Family history of obesity			
Yes	3 (25.0)	9 (75.0)	0.728
No	14 (35.9)	25 (64.1)	
Waist circumference			
≤ 88 cm	17 (56.7)	13 (43.3)	<0.001*
> 88 cm	0 (0.00)	21 (100)	
Total free sugar consumption			
< 25 g/d	0 (0.00	4 (100)	0.288
≥ 25 g/d	17 (36.2)	30 (63.8)	
Smoking status			
Yes	3 (50.0)	3 (50.0)	0.387
No	14 (31.1)	31 (68.9)	
Supplement use			
Yes	5 (50.0)	5 (50.0)	0.270
No	12 (29.3)	29 (70.7)	
Physical activity			
None	4 (26.7)	11 (73.3)	0.267
Once per week	1 (11.1)	8 (88.9)	
2-3 times per week	9 (50.0)	9 (50.0)	
4-5 times per week	0 (0.00)	1 (100)	
Daily	3 (37.5)	5 (62.5)	

*Significant at 95% confidence level. Numbers in the table are frequency (%). Fisher’s Exact test was performed to obtain the data provided in the table.

### 3.3 Influence of weight status and lifestyle habits on the proportion of iNKT cells

**Figure 1:**
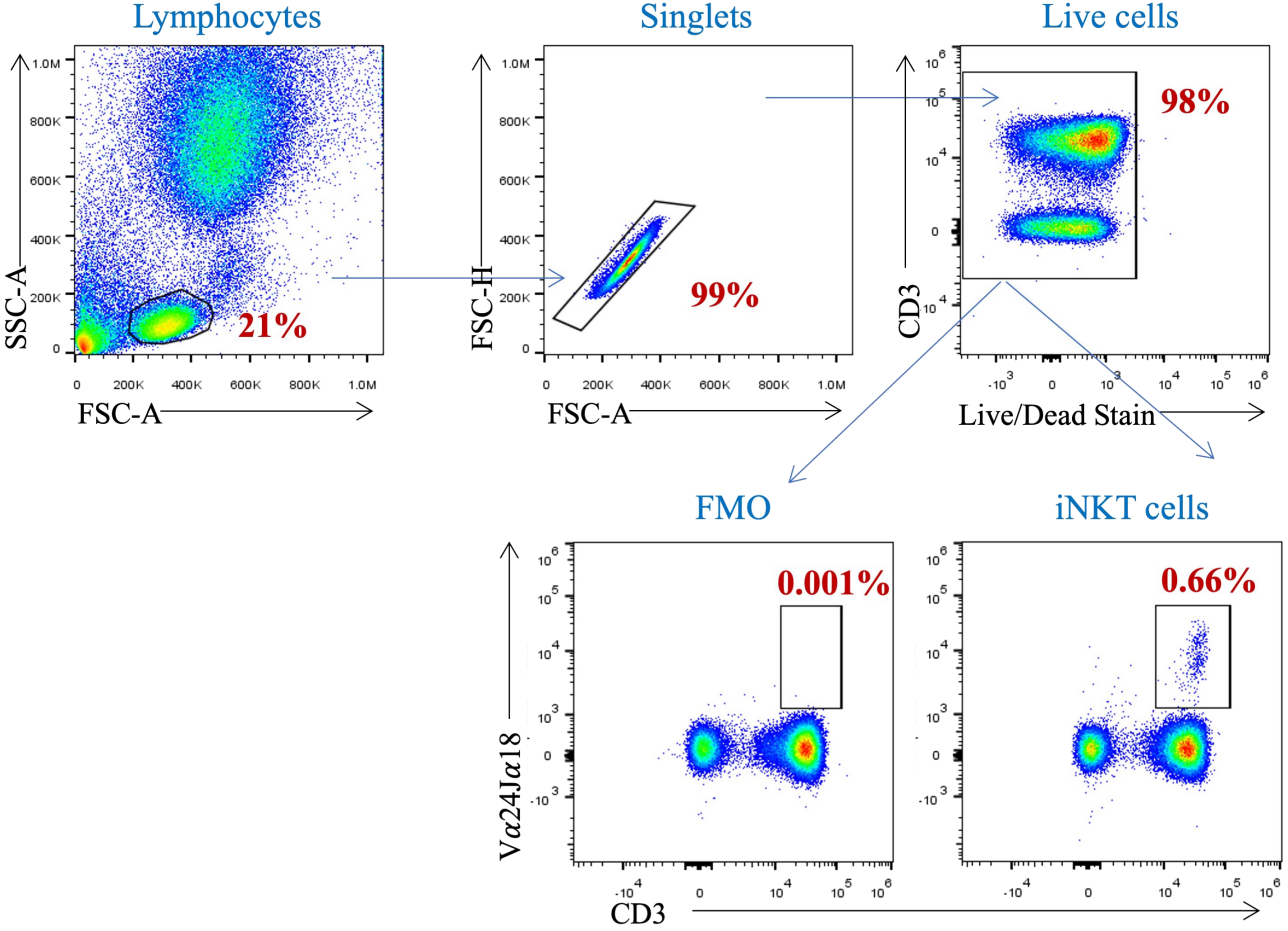
Gating Strategy for the identification of iNKT cell population in peripheral blood. PBMCs isolated from whole blood were stained with anti-CD3, anti-CD4 and anti-Vα24Jα18 mAbs. Cells were pre-gated on live, single, and CD4^+^ cells before identifying the iNKT cells population (CD3^+^ Vα24Jα18^+^ cells).

**Figure 2:**
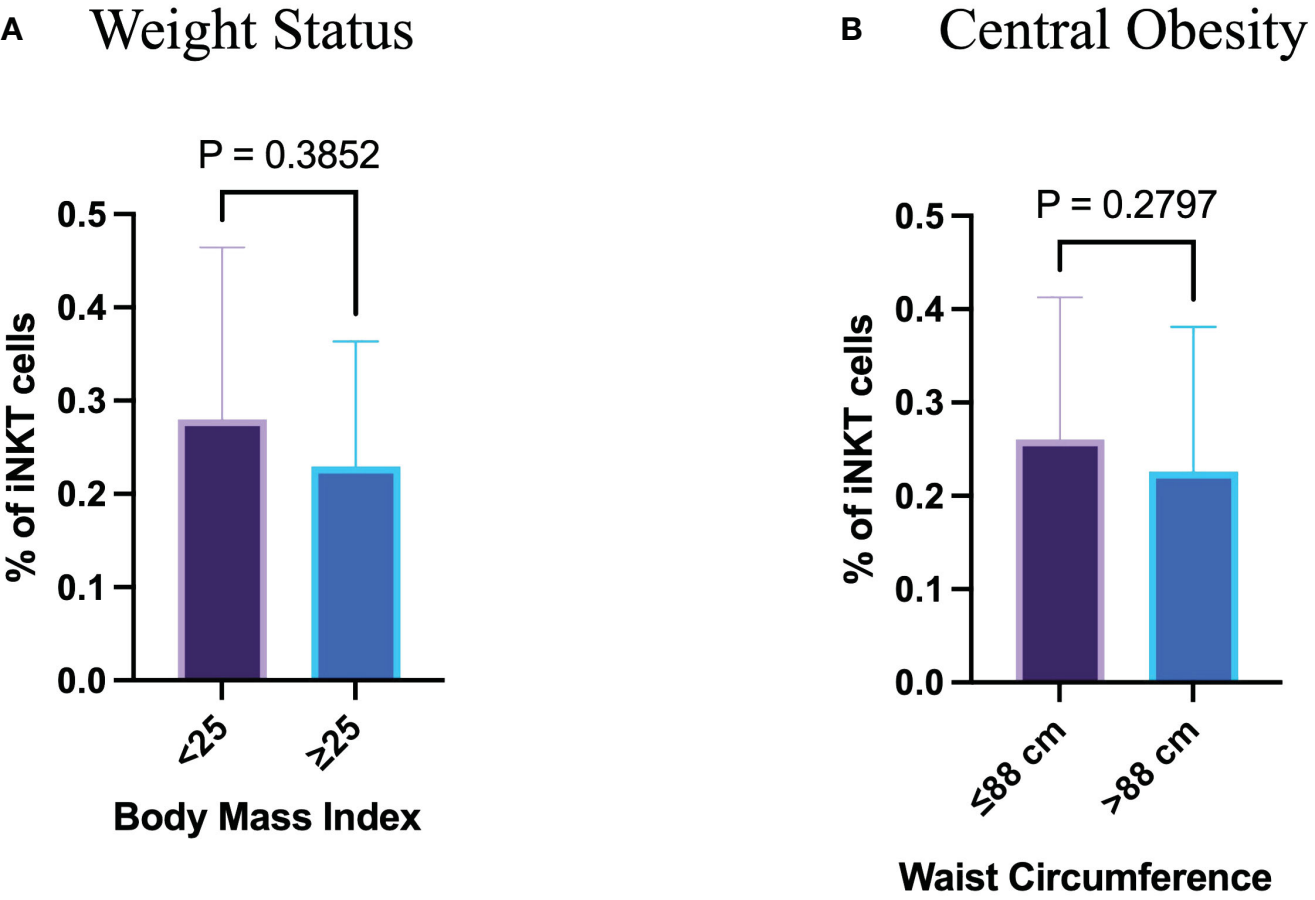
Neither weight status nor waist circumference have influences on peripheral iNKT cells. **(A)** Percentage of iNKT cells based on body mass index. **(B)** Percentage of iNKT cells based on waist circumference.

To investigate the influence of free sugar intake on the proportion of circulating iNKT cells, a gating strategy were set up to identify such cells in the participants’ peripheral blood samples. Using the surface markers; CD3, CD4 and Vα24Jα18 TCR, we identified the iNKT cells among the PBMCs (**Figure 1**). The proportion of peripheral iNKT cells was evaluated according to the BMI and WC. Neither the participants with a BMI ≥ 25 kg/m^2^ nor those with a WC > 88 cm showed a statistical difference in the proportion of circulating iNKT cells comparing to lean healthy participants (*p*= 0.385 and *p*= 0.280, respectively) (**Figures 2A, B**).

**Figure 3:**
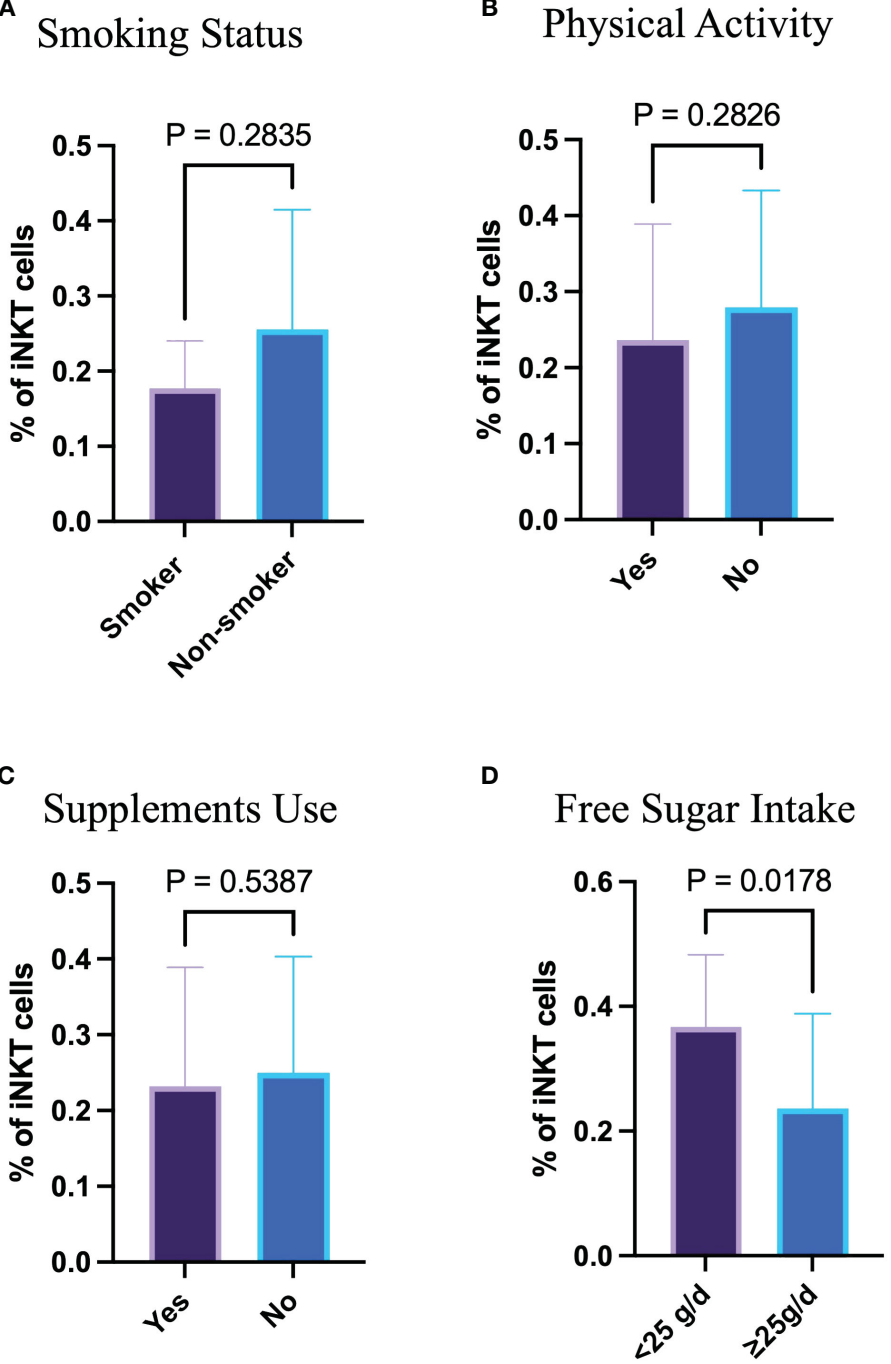
High sugar intake affects the frequency of circulating iNKT cells. **(A)** Percentage of iNKT cells based on smoking status. **(B)** Percentage of iNKT cells based on physical activity. **(C)** Percentage of iNKT cells based on supplement use. **(D)** Percentage of iNKT cells based on free sugar intake.

The association between lifestyle habits such as physical activity, smoking status, supplement use, and free sugar intake in relation to the proportion of circulating iNKT cells was assessed. Smokers have similar proportion of circulating iNKT cells compared with non-smokers, *p*= 0.284 (**Figure 3A**). Having said that, only 6 participants out of 50 were smokers. Additionally, physical activity and supplement use were not linked to the proportion of iNKT cells (*p*= 0.283 and *p*= 0.539, respectively) (**Figures 3B, C**). However, excessive free sugar consumption above the recommendation (≥ 25 g/day) was associated with the smaller proportion of circulating iNKT cells (*p*= 0.018) (**Figure 3D**). These findings indicate that high free sugar consumption may influence the immune function.

### 3.4 Association between the proportion of peripheral iNKT cells and free sugar intake based on weight status

Spearman correlation was conducted to assess the strength of the association between free sugar intake and the proportion of circulating iNKT cells among participants experiencing overweight and obesity. Results indicated a significant moderate correlation between the proportion of iNKT cells and free sugar intake from solid food sources and total free sugar intake only among the overweight and obese participants (r_s_= -0.612 and r_s_= -0.523, respectively). Neither the BMI nor the WC was correlated with the proportion of iNKT cells in participants experiencing overweight and obesity (**Table 3**). In contrast, this was not the case among the healthy participants, as the BMI was negatively correlated with the proportion of iNKT cells (r_s_= -0.547, *p*< 0.05). However, when considering all participants BMI was not correlated with iNKT cells (r_s_= -0.186, *p*= 0.192)

**Table 3:** Correlation between frequencies of peripheral CD3^+^, CD4^+^ and iNKT cells and body mass index and free sugar intake.

	% of CD3^+^ cells	% of CD3^+^ CD4^+^ cells	% of iNKT cells
Healthy weight women (n= 17)			
Body mass index, Kg/m^2^	r_s_=0.071 *p*=0.787	r_s_=-0.013 *p*=0.960	r_s_=0.5470* *p*=0.0248
Waist circumference, cm	r_s_=0.025 *p*=0.923	r_s_=0.037 *p* =0.886	r_s_=-0.320 *p* =0.209
Total free sugar, g/d	r_s_=-0.083 *p* =0.751	r_s_=-0.206 *p* =0.424	r_s_=0.009 *p* =0.975
Free sugar from liquid food sources, g/d	r_s_=-0.219 *p* =0.396	r_s_=-0.231 *p* =0.368	r_s_=0.015 *p=*0.955
Free sugar from solid food sources, g/d	r_s_=0.071 *p* =0.787	r_s_=-0.115 *p* =0.657	r_s_=-0.037 *p* =0.887
Women experiencing overweight and obesity (n= 34)			
Body mass index, Kg/m^2^	r_s_=-0.072 *p*=0.684	r_s_=0.099 *p*=0.578	r_s_=0.055 *p*=0.757
Waist circumference, cm	r_s_=-0.114 *p* =0.517	r_s_=-0.095 *p* =0.591	r_s_=-0.183 *p* =0.300
Total free sugar, g/d	r_s_=0.136 *p* =0.443	r_s_=0.079 *p* =0.658	r_s_ =-0.523* *p* **=0.0015**
Free sugar from liquid food sources, g/d	r_s_=0.263 *p* =0.132	r_s_=0.014 *p* =0.935	r_s_=-0.063 *p* =0.723
Free sugar from solid food sources, g/d	r_s_=-0.015 *p* =0.923	r_s_=0.134 *p* =0.449	r_s_ =-0.612* *p* **=0.0001**

*Significant at 95% confidence level.

**Figure 4:**
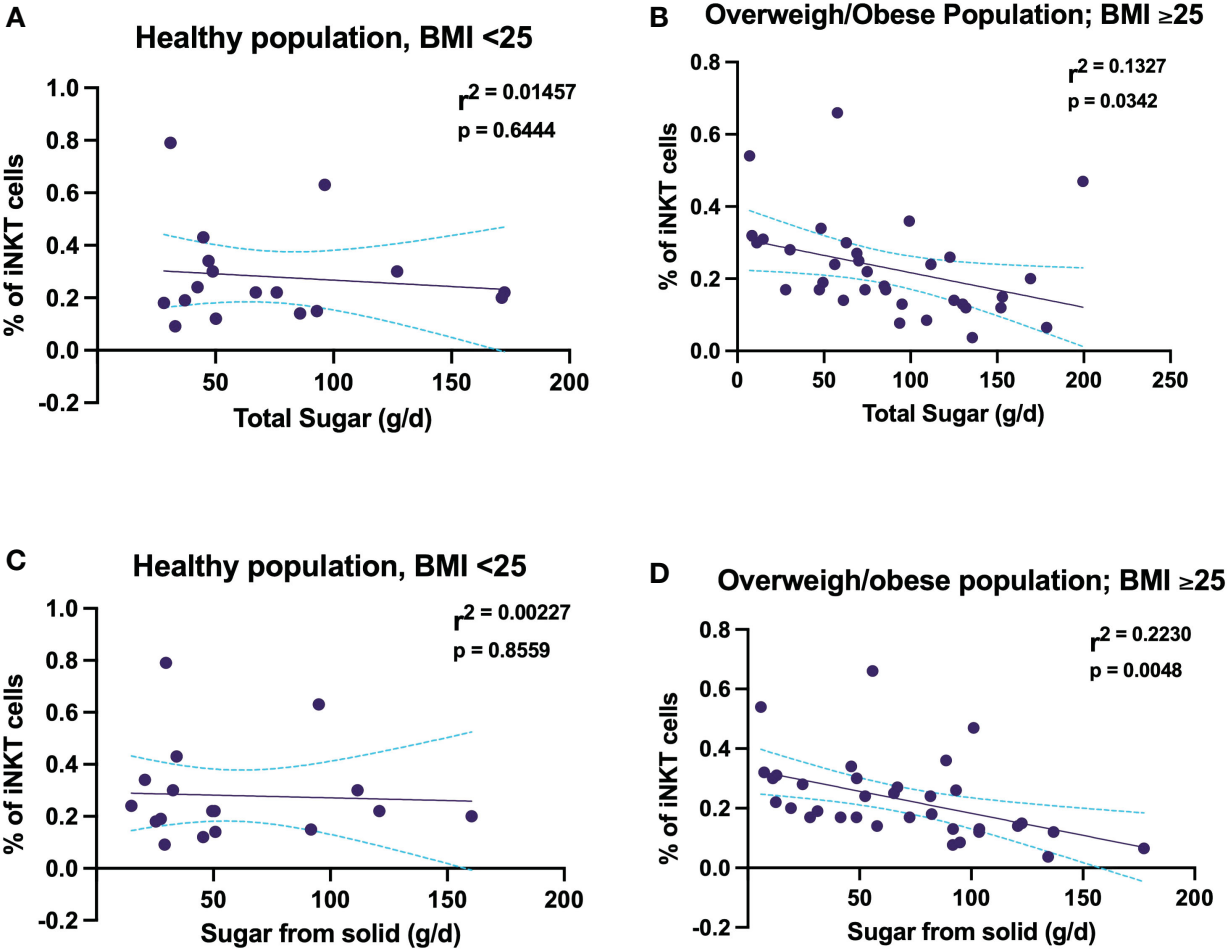
Free sugar intake is associated with decreasing the proportion of circulating iNKT cells in women experiencing overweight and obesity. **(A)** Association between percentage of iNKT and total free sugar in healthy women. **(B)** Association between percentage of iNKT and total free sugar in women experiencing overweight and obesity. **(C)** Association between percentage of iNKT and free sugar coming from solid food sources in healthy women. **(D)** Association between percentage of iNKT and free sugar coming from solid food sources in women experiencing overweight and obesity.

Healthy weight participant showed that there was no association with total sugar intake and sugar coming from solid sources and the proportion of iNKT cells (**Figures 4A, C**). In contrast, among the overweight and obese participants total free sugar intake predicted a smaller proportion of iNKT cells frequency, wherein total free sugar intake explained 13% of the change in proportion of iNKT cells (**Figure 4B**). Moreover, the correlation between sugar intake from solid food sources and the proportion of iNKT cells were only evident among the participants with a BMI of ≥ 25 kg/m^2^ (**Figure 4D**). These findings show that high free sugar intake may affect the proportion of peripheral iNKT cells, consequently leading to immune dysfunction.

Simple linear regression analysis was performed to explore the association between the proportion of circulating iNKT cells and free sugar intake adjusting for weight status (**Table 4**). Results showed significant association between proportion of circulating iNKT cells and total free sugar intake and free sugar intake coming from solid food sources, but not free sugar intake coming from liquid food sources, only among overweight and obese women (Beta: -0.10: Standard Error: 0.04 [95% Confidence Interval: -0.18 to -0.01], *p*= 0.034) and (Beta: -0.15: Standard Error: 0.05 [95% Confidence Interval: -0.25 to -0.05], *p*= 0.005), respectively. These two models explained 13% and 22% of the change occurred in the proportion of circulating iNKT cells with high consumption of total free sugar and free sugar coming from solid food sources, respectively.

**Table 4:** Simple linear regression analysis between the proportion of iNKT cells and free sugar intake among healthy weight women and women experiencing overweight and obesity.

Variable	Beta	Standard Error	*p-*value	95% Confidence Interval	R-squared
Healthy weight women (n= 17)					
Total free sugar, 100 g/d	-0.05	0.10	0.644	-0.27 to 0.17	0.02
Free sugar from liquid food sources, 100 g/d	-0.33	0.33	0.330	-1.03 to 0.37	0.06
Free sugar from solid food sources, 100 g/d	-0.02	0.11	0.856	-0.26 to 0.22	0.00
Women experiencing overweight and obesity (n= 34)					
Total free sugar, 100 g/d	-0.10	0.04	0.034*	-0.18 to -0.01	0.13
Free sugar from liquid food sources, 100 g/d	0.02	0.08	0.763	-0.13 to 0.18	0.00
Free sugar from solid food sources, 100 g/d	-0.15	0.05	0.005*	-0.25 to -0.05	0.22

*Significant at 95% confidence level.

## 4 Discussion

This study was set out to explore the effect of lifestyle habits such as smoking status, physical activity, supplement intake and free sugar consumption on the proportion of iNKT cells and the correlation with weight status. Although there was a trend in decreasing the proportion of circulating iNKT cells among the overweight and obese participants comparing to healthy participants, the reduction was insignificant. The importance of iNKT cells in obesity development has been emphasized in a number of previously published reports. The trend of decreasing the proportion of peripheral iNKT cell is consistent with others. For example, Carolan et al. reported that obese children showed a reduced proportion of circulating iNKT cells associated with insulin resistance (40). Moreover, Lynch, et al. found that the proportion of omental iNKT cells was smaller in their obese population than in their heathy controls (41). iNKT cells have shown a protective role against diet induced obesity and other metabolic disorders induced by diet through the production of anti-inflammatory cytokines such as IL-4 and IL-10 (42). Restoring the activity of iNKT cells in obese mice has been reported to increase weight loss and decrease insulin tolerance (43, 44). These findings suggest that obesity has a direct effect on the proportion of iNKT cells, which may induce insulin resistance and other metabolic disorders. Notably, the sample size of our study was relatively small. Further studies involving a larger and more diverse population, including both men and women, are needed to evaluate the significance of iNKT cells and their correlation with weight status. This could lead to the development of a novel immunotherapy to combat obesity and prevent metabolic disorders.

Previous research reported a negative impact of smoking on the proportion and function of circulating iNKT cells by impairing cytokines secretion (45). In contrary, Ström, et al. has reported that smokers had a remarkably increased proportion of iNKT cells compared with non-smokers (46). In this study, smoking was not associated with the proportion of circulated iNKT cells. Having said that, only 6 participants out of 50 were smokers among both healthy and overweight/obese participants. These inconsistent findings regarding the effect of smoking on iNKT cells can be explained by the different sample sizes included in these studies as well as sample characteristics such as sex, weight status, and many other factors.

In the present study, free sugar consumption was significantly associated with reduced proportion of circulating iNKT cells. A number of previous studies have examined the effect of high sugar diet on other immune cells. For example, a study found that increased intake of artificial sweeteners such as sucralose negatively influenced CD8^+^ T cells (47). Mice treated with large amounts of sucralose showed CD8^+^ T cell dysfunction among mice challenged with Listeria monocytogenes or murine models of cancer (47). Moreover, Zhang et al. revealed the influence of high sugar consumption on exacerbating autoimmune diseases, such as colitis and experimental autoimmune encephalomyelitis by promoting the pathogenesis of Th17 cells (23). In another study, Th17 cells were depleted in mice that were fed Western-style high-fat diet, promoting the development of metabolic syndrome (48). Collectively, increased consumption of free/added sugar can negatively influence the immune function, ultimately contributing to the development of inflammatory diseases and metabolic disorders.

A negative association between free sugar consumption (total free sugar and free sugar from solid food sources) and the proportion of circulating iNKT cells was observed only among the overweight and obese participants in this study. This observation could be attributed to the fact that individuals experiencing overweight and obesity often have different eating habits, variations in adipocyte characteristics, hormonal status, genetic factors, and metabolic processes (49, 50). These differences may lead to distinct responses to varying levels of sweet cravings and consequently free sugar consumption. It has been shown that a high-sugar diet and an increased BMI can interrupt the gut microbiome, which may have a negative consequence on immune cell development (51, 52). This could explain why we found a reduced proportion of iNKT cells in the participants with a BMI ≥ 25 kg/m^2^ and that such proportion was negatively correlated with free sugar intake. Since iNKT cells recognize glycolipids presented by CD1d molecules (26), altering the microbiome composition can influence the proportion and function of iNKT cells. In a previous study, specific pathogen-free mice showed an impaired function of iNKT cells compared with control mice (53), highlighting the importance of mucosal microbiome composition among iNKT cells. Taken together, these findings suggest that high free sugar consumption may alter the microbiota, ultimately affecting immune function.

This study is the first to assess a number of life-style habits including free sugar consumption, smoking, and physical activity level in relation to the proportion of circulating iNKT cells based on weight status. However, this study is limited by the inclusion of women only which result in limited generalizability of study findings. Future research should be conducted among women and men to compare differences across the groups. Additionally, including a larger and more diverse population would extend the overall view of the harmful effects of unhealthy lifestyle habits on iNKT cells. The current study also assessed only the proportion of iNKT cells; this measurement may not accurately reflect the actual number of these cells.

## 5 Conclusions

A fast-paced lifestyle has introduced various unhealthy eating habits, such as the excessive consumption of highly processed sugary foods, which can contribute to the development of inflammatory and chronic diseases as well as metabolic disorders, consequently affecting overall health and quality of life. Understanding the interplay between the proportion of iNKT cells and lifestyle factors such as free sugar consumption, smoking, and physical activity in relation to weight status could help in tailoring new strategies that aim to raise awareness of unhealthy lifestyle habits and therefore preventing obesity and other metabolic disorders.

## Data availability statement

The original contributions presented in the study are included in the article/supplementary material. Further inquiries can be directed to the corresponding author.

## Ethics statement

The studies involving humans were approved by Ethical approval for the study was obtained from the Scientific Research and Ethics Committee of the College of Applied Medical Sciences, Taibah University, Madinah (project number; 2023/154/105 MLT). The studies were conducted in accordance with the local legislation and institutional requirements. The participants provided their written informed consent to participate in this study.

## Author contributions

ReA: Conceptualization, Formal analysis, Funding acquisition, Investigation, Methodology, Resources, Software, Supervision, Validation, Writing – original draft, Writing – review & editing. YA: Formal analysis, Methodology, Writing – review & editing. BA: Investigation, Methodology, Writing – review & editing. JA: Investigation, Methodology, Writing – review & editing. MA: Investigation, Methodology, Writing – review & editing. NA: Investigation, Methodology, Writing – review & editing. RaA: Investigation, Methodology, Writing – review & editing. WaA: Investigation, Methodology, Writing – review & editing. WeA: Investigation, Methodology, Writing – review & editing. HE: Formal analysis, Methodology, Writing – review & editing. WM: Formal analysis, Methodology, Writing – review & editing.
